# Parental EV-A71 vaccine hesitancy amid shifting HFMD pathogens: a cross-sectional study in Guangzhou

**DOI:** 10.1186/s12889-026-29951-0

**Published:** 2026-10-19

**Authors:** Xin Su, Zhixiong Gao, Jiewei Zhong, Conghua Bai, Ruili Xie, Lei Luo, Qilin Wu

**Affiliations:** 1 Guangzhou Field Epidemiology Training Program, No.1 Qide Road, Guangzhou, Guangdong 510440 China; 2 Guangzhou Panyu Center for Disease Control and Prevention (Guangzhou Panyu Health Supervision Institute), No.71 Desheng Road, Panyu District, Guangzhou, Guangdong 511400 China; 3 Guangzhou Huadu Center for Disease Control and Prevention (Guangzhou Huadu Health Supervision Institute), No.14 Gongyi Road, Huadu District, Guangzhou, Guangdong 510800 China; 4 Guangzhou Tianhe Center for Disease Control and Prevention (Guangzhou Tianhe Health Supervision Institute), No.15 Keyun Middle Road, Tianhe District, Guangzhou, Guangdong 510000 China; 5 Guangdong Provincial Center for Disease Control and Prevention, No.160 Qunxian Road, Panyu District, Guangzhou, Guangdong 510000 China; 6 Guangzhou Center for Disease Control and Prevention (Guangzhou Health Supervision Institute), No 1, Qide Road, Guangzhou, Guangdong 510440 China

**Keywords:** EV-A71 vaccine, Vaccination hesitancy, Structural Equation Modeling, Parents of children, Influencing factors

## Abstract

**Background:**

Following enterovirus A71 (EV‑A71) vaccine introduction, the hand foot mouth disease (HFMD) pathogen spectrum has shifted away from EV‑A71, prompting parental doubts about the vaccine’s continued relevance. This study aimed to investigate the prevalence and determinants of EV‑A71 vaccine hesitancy among parents in Guangzhou.

**Methods:**

We surveyed parents of children ≤ 5 years at 10 community health centers in Guangzhou using stratified multi-stage sampling. Exploratory factor analysis identified latent variables, followed by structural equation modeling (SEM) to examine their direct and indirect effects on vaccine hesitancy. Logistic regression with cluster-robust standard errors was used to assess the effects of parental knowledge, attitude, and practice (KAP) and physician recommendation practices, specifically examining the cross-level interaction between physician recommendation enthusiasm and parental vaccine confidence.

**Results:**

The prevalence of EV-A71 vaccine hesitancy among parents was 28.95%. Mothers (OR: 0.46[0.28, 0.76]) and parents with a college degree or associate degree (OR: 0.53[0.32, 0.86]) were less hesitant, whereas being a non‑healthcare worker (OR: 2.38[1.03, 5.55]) and having a child without a history of HFMD (OR: 3.45[1.31, 9.12]) were more hesitant. SEM indicated that vaccine confidence (*β* = -0.39), service trust (*β* = -0.17), and risk perception (*β* = -0.17) exerted negative direct effects on vaccine hesitancy. Knowledge had no direct effect, its total indirect effect via the three mediators was significant(*β* = -0.45). Furthermore, the interaction between parental confidence and physician recommendation was marginally significant(*β* = 2.10, *P* = 0.08).

**Conclusion:**

Knowledge reduces hesitancy mainly through confidence, trust, and risk perception, not directly. Communication and counseling addressing these attitudes may support informed parental decisions about voluntary, self-paid EV-A71 vaccination.

**Supplementary Information:**

The online version contains supplementary material available at https://doi.org/10.1186/s12889-026-29951-0.

## Introduction

Hand, foot, and mouth disease (HFMD) is a highly contagious infectious disease that predominantly affects children under 5 years of age. With multiple transmission routes, it consistently ranks among the highest incidence rates of Class C notifiable infectious diseases in China [1]. HFMD is caused by various enterovirus serotypes, including enterovirus A71 (EV-A71, coxsackievirus A16 (CV-A16), coxsackievirus A6 (CV-A6), and coxsackievirus A10 (CV-A10), and these serotypes confer no cross-immunity [2, 3]. Among these, EV-A71 is the primary pathogen responsible for severe, critical, and fatal cases, capable of causing encephalitis, myocarditis, and neurogenic pulmonary edema, thereby posing a significant threat to infants and young children [3, 4].

In China, the inactivated EV-A71 vaccine was introduced in 2016 for the prevention of EV-A71-related HFMD. It is a self-paid, non-National Immunization Program vaccine, and is generally recommended for children aged 6 months to 5 years as a two-dose series administered one month apart, preferably completed before 12 months of age. Parents usually obtain relevant information through channels such as the preventive health care departments of community health service centers, maternal and child health services, and kindergartens. Previous estimates suggest, between 2008 and 2015, the annual costs (direct medical expenses and indirect costs) associated with severe and mild HFMD cases caused by EV-A71 infection in China were 180 million yuan and 1–2 billion yuan or more, respectively [5]. Following the widespread adoption of the vaccine, a real-world study has estimated that EV-A71 vaccination averted approximately 300,000 HFMD cases between 2017 and 2018 [6], which equates to savings of approximately 240–420 million yuan in the total socioeconomic burden [7]. Following the rollout of vaccination, however, the etiological spectrum of HFMD has shifted markedly from a predominance of EV-A71 and CV-A16 toward other serotypes, such as CV-A6 and CV-A10 [8]. Surveillance data from Guangzhou indicate that CV-A6 became the dominant strain during 2015–2023, while the detection prevalence of EV-A71 declined significantly [9]. This epidemiological shift has inadvertently dampened parents’ willingness to vaccinate, fostering a parental perception that the vaccine is less relevant, thereby undermining recognition of its core benefit in preventing severe disease and death.

Vaccine hesitancy is defined as delayed acceptance or refusal of vaccination despite available services [10]. Prevalence of EV-A71 vaccine hesitancy has varied, from 33% in Jinan [11] to nearly 50% in Chaozhou [12], and hesitancy is not static; a longitudinal Coronavirus Disease 2019 (COVID-19) study found that 37% of initially hesitant individuals later became willing [13]. Physicians’ communication and recommendations may influence parents’ decisions about voluntary, self-paid vaccine [14–15], including EV-A71, which is not in the National Immunization Program. EV-A71 vaccination was initially used mainly in mainland China, but in 2026, Vietnam and Malaysia approved EV-A71 vaccines following recurrent HFMD outbreaks and substantial local burden [16, 17]. These approvals reflect national-level decisions shaped by local disease burden; however, whether children actually get vaccinated still depends on parental acceptance. This study therefore focuses on parental hesitancy toward a licensed, voluntary, self-paid EV-A71 vaccine in Guangzhou, China, rather than on evaluating global public health need or cost-effectiveness. Specifically, this study aimed to investigate the prevalence of EV-A71 vaccination hesitancy among parents of children under 5 years of age in Guangzhou, and to explore the direct and indirect pathways through which parental knowledge, attitudes, and practices (KAP) influence hesitancy, as well as the role of community physicians’ communication and recommendations in shaping parental vaccination decisions.

## Methods

### Study participants and sampling

We stratified Guangzhou’s 11 districts into three zones (central urban area, urban‑rural fringe, and outer integrated zone) based on the Guangzhou Urban Master Plan (2017–2035), then randomly picked one district (Liwan, Panyu, and Huadu) from each and ten community health centers (CHCs) within them.

Data collection was conducted in April 2026. Parents accompanying children aged ≤ 5 years to the selected CHCs for medical care or vaccination were recruited via convenience sampling. Eligible parents were the primary caregivers of at least one child aged ≤ 5 years and were long-term residents of Guangzhou. Long-term residents were defined as parents or guardians who had resided continuously in Guangzhou for at least 3 months, including both registered and non-registered residents, in accordance with the National Basic Public Health Service Program. Parents were excluded if their child had medical contraindications to vaccination (e.g., immunodeficiency or neurological disorders), or if they were not the primary decision-makers for the child’s vaccination (e.g., nannies or distant relatives).Additionally, healthcare workers responsible for vaccination services at the same 10 centers were invited to participate to evaluate the provider perspective.

The sample size was calculated using the single proportion formula: *n=Z*^*2*^*P(1 − P)/d*^*2*^. Assuming a 95% confidence level (*Z* = 1.96), an anticipated vaccine hesitancy prevalence of 35% based on previous literature [11], and a margin of error of 5% (*d* = 0.05), the initial estimated sample size was approximately 350 participants. After adjusting for a 10% non-response rate, the target sample was set at a minimum of 385 parents.

The study protocol was approved by the Ethics Committee of Guangzhou Center for Disease Control and Prevention (Guangzhou Health Supervision Institute). All participants provided written informed consent before participation.

### Data collection

Participants completed the questionnaire independently, with a trained investigator available for clarification (The full questionnaire is available in Supplementary File 1.). The questionnaire captured demographic characteristics, knowledge about HFMD and the EV-A71 vaccine (11 items, score 0–11), and attitudes toward the disease and vaccine, including risk perception, vaccine confidence, and trust in services, rated on a 5-point Likert scale. Healthcare workers completed an online questionnaire assessing their recommendation practices, professional knowledge, vaccine confidence, and recommendation enthusiasm (see supplementary file 2).

Vaccine hesitancy was defined as a “No” or “Uncertain” response to the question: “Are you willing to have your child vaccinated against EV-A71?” The response “Yes” (already vaccinated or planning to vaccinate) was classified as non‑hesitant, consistent with the WHO definition of vaccine hesitancy [10].

### Statistical analysis

Analyses used R 4.5.3. Chi‑square tests and t‑tests were used for univariate comparisons between hesitant and non-hesitant groups. Variables with *P* < 0.05 were entered into a multivariable logistic regression to identify sociodemographic determinants. Principal component analysis with varimax rotation extracted latent factors from the parental attitude questionnaire, and a structural equation model (SEM) was constructed based on the Health Belief Model to examine direct and indirect pathways from knowledge, vaccine confidence, service trust, and risk perception to vaccine hesitancy. The model was estimated using maximum likelihood. Additionally, logistic regression models with cluster‑robust standard errors were applied to assess the effects of parental KAP and physician recommendation practices on hesitancy, including the cross‑level interaction between physicians’ recommendation enthusiasm and parental vaccine confidence. Statistical significance was set at *P* < 0.05.

## Results

### Demographic characteristics and EV-A71 vaccine hesitancy

A total of 445 parents completed the survey. Thirty-four were excluded because their child was outside the eligible age range (≤ 5 years), leaving 411 participants for analysis. The prevalence of vaccine hesitancy was 28.95% (119/411). Fathers, less-educated parents, non-healthcare worker parents, and those whose child had no prior HFMD had significantly higher hesitancy prevelance in bivariate comparisons (all *P* < 0.05) (Table 1).

**Table 1:** Comparison of parental EV-A71 vaccine hesitancy for children by demographic characteristics among 411 respondents

Content	Participants, *n* (%)	Hesitant cases	Prevalence of vaccine hesitancy(%)	*χ* ^2^	*P* value
Relationship with child				12.35	**0.006**
Father	89(21.65)	39	43.82		
Mother	312(75.91)	78	25.00		
Grandparent(s)	10(2.43)	3	30.00		
Parental education				9.63	**0.008**
High school or below	102(24.82)	42	41.18		
College or associate degree	294(71.53)	75	25.51		
Master’s degree or above	15( 3.65)	3	20.00		
Occupation				6.74	**0.009**
Healthcare worker	51(12.41)	7	13.73		
Non-healthcare worker	360(87.59)	113	31.39		
Average monthly household income (CNY)				1.33	0.514
< 5,000	125(30.41)	36	28.80		
5,001–10,000	141(34.31)	37	26.24		
> 10,000	145(35.28)	47	32.41		
Child’s history of HFMD^#^				6.77	**0.009**
Yes	42(10.22)	5	11.90		
No	369(89.78)	115	31.17		

Bold values indicate statistically significant differences at *P* < 0.05

^#^: Child’s history of HFMD was recorded for all children, regardless of their EV-A71 vaccination status

### Factor analysis of parental attitudes toward HFMD and EV-A71 vaccine

Exploratory factor analysis was performed using principal component analysis (PCA) with varimax orthogonal rotation on nine items assessing parental attitudes toward the disease and vaccine. The Kaiser-Meyer-Olkin (KMO) measure was 0.84, and Bartlett’s test of sphericity was significant( *χ²* =982.29, *P* < 0.001), indicating that the data were suitable for PCA. After varimax rotation, three factors with eigenvalues greater than 1.0 were extracted, with eigenvalues of 3.14, 2.19, and 1.73, respectively, accounting for 28.57%, 19.90%, and 15.74% of the variance(cumulative 64.22%). Factor 1, labeled “vaccine confidence,” comprised four items; Factor 2, labeled “services trust,” comprised three items; and Factor 3, labeled “perceived risk,” comprised two items (Table 2).

**Table 2:** Factor loadings of items on attitudes toward HFMD and EV-A71 vaccine after varimax rotation

Item	Factor 1	Factor 2	Factor 3
Perceived risk of HFMD infection in children under 5 years in Guangzhou			0.73
Concern about severe disease due to HFMD infection in their child			0.79
Belief that the EV-A71 vaccine is safe	0.74		
Belief that the EV-A71 vaccine can prevent HFMD	0.80		
Belief that the EV-A71 vaccine can reduce severe cases and deaths from HFMD	0.78		
Acceptance of possible HFMD infection despite vaccination	0.76		
Trust in official vaccine-related information		0.70	
Trust in vaccination providers		0.82	
Agreement that providers recommend vaccines primarily for children’s health, rather than to meet quotas or generate revenue		0.79	

### Comparison of factor scores between groups

We used an independent samples t-test to analyze differences between groups in cognitive scores (calculated from knowledge items) and scores on the three factors (derived from PCA) and found that the hesitant group scored significantly lower than the non-hesitant group on cognition, vaccine confidence, service trust, and risk perception (all *P* < 0.001) (Table 3).

**Table 3:** Comparison of cognition, vaccine confidence, service trust, and risk perception scores between hesitant and non-hesitant groups

Variable	Non-hesitant (Mean ± SD)	Hesitant (Mean ± SD)	t	*P* value
Cognition score	4.65 ± 1.96	3.01 ± 2.29	7.33	**< 0.001**
Vaccine confidence score	15.67 ± 2.48	12.98 ± 2.28	10.23	**< 0.001**
Service trust score	12.77 ± 1.70	11.17 ± 2.04	6.08	**< 0.001**
Risk perception score	8.37 ± 1.36	7.55 ± 1.47	5.42	**< 0.001**

Bold values indicate statistically significant differences at *P* < 0.05

### Sociodemographic determinants of EV-A71 vaccine hesitancy

Variables significant in univariate chi‑square tests (child relationship, parental education, occupation, child HFMD history) were entered into multivariable logistic regression.The results revealed that being the mother (OR: 0.46[0.28, 0.76], *P* = 0.003) and having a college or associate degree (OR: 0.53[0.32, 0.86], *P =* 0.010) were protective factors against vaccine hesitancy, whereas being a non-healthcare worker (OR: 2.38[1.03, 5.55], *P* = 0.044) and having a child without a history of HFMD (OR: 3.45[1.31, 9.12], *P =* 0.012) were risk factors (Table 4).

**Table 4:** Multivariable logistic regression of sociodemographic determinants of EV-A71 vaccine hesitancy

Variable	OR	95% CI	*P* value
Relationship with child			
Father (reference)	1.00		
Mother	0.46	0.28–0.76	**0.003**
Grandparent(s)	0.39	0.09–1.70	0.209
Parental education level			
High school or below (reference)	1.00		
College or associate degree	0.53	0.32–0.86	**0.010**
Master’s degree or above	0.36	0.10–1.38	0.136
Occupation			
Healthcare worker (reference)	1.00		
Non-healthcare worker	2.38	1.03–5.55	**0.044**
Child’s history of HFMD			
Yes (reference)	1.00		
No	3.45	1.31–9.12	**0.012**

Bold values indicate statistically significant differences at *P* < 0.05

### SEM of pathways to EV-A71 vaccine hesitancy

**Fig. 1:**
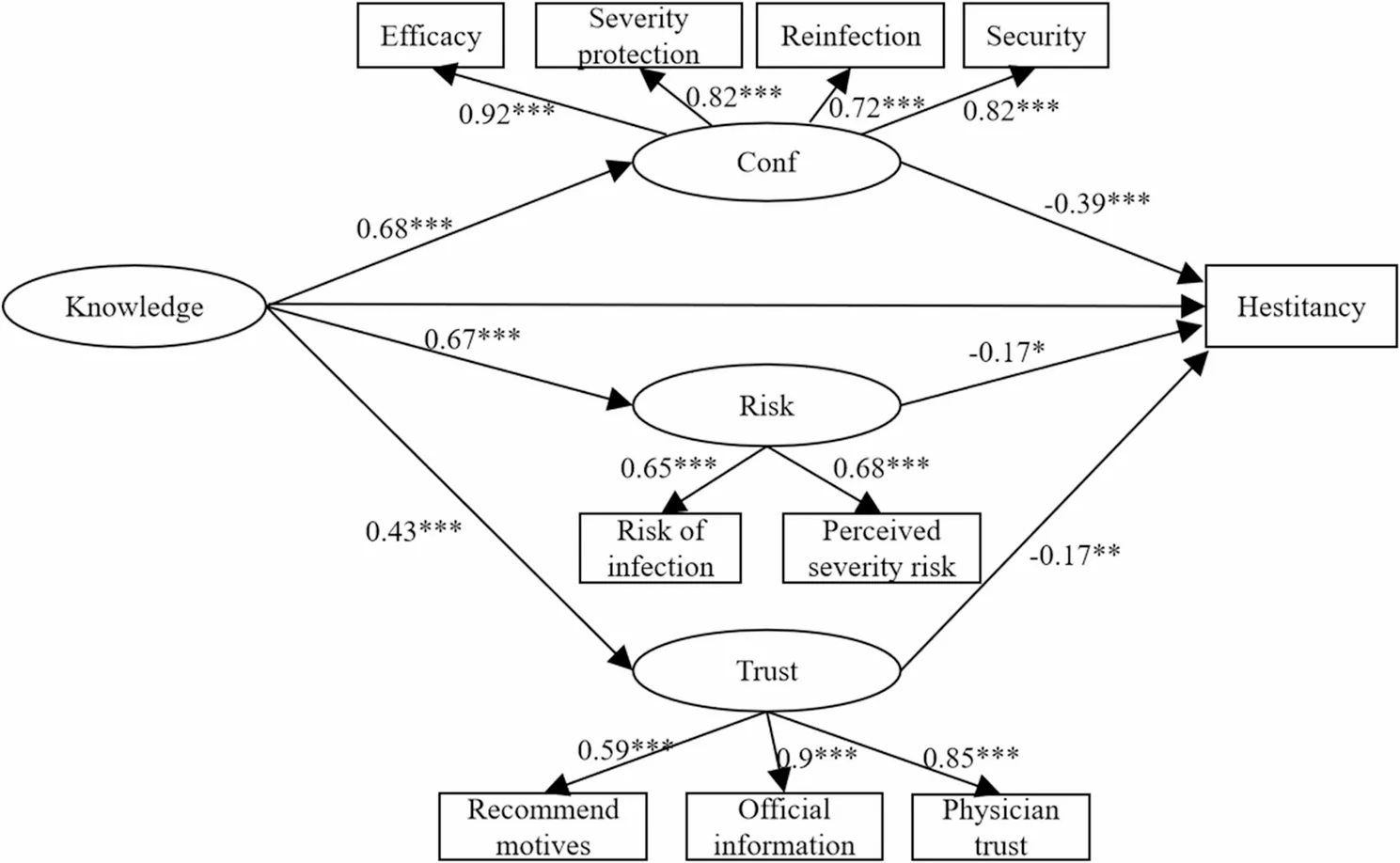
SEM examining the direct and indirect pathways of HBM constructs on EV-A71 vaccine hesitancy among parents of children aged 5 years and below in Guangzhou. Standardized path coefficients are shown on the arrows. *<0.05, **<0.01, ***<0.001

Vaccine confidence, service trust, and risk perception were specified as exogenous latent variables, with their constituent items serving as observed indicators, and EV-A71 vaccine hesitancy was designated as the endogenous latent variable. The model was estimated using maximum likelihood. Vaccine confidence (*β*= -0.39, *P* < 0.001), service trust (*β*=-0.17, *P =* 0.015), and risk perception (*β*=-0.17, *P* = 0.011) all exerted significant negative direct effects on vaccine hesitancy. The direct effect of knowledge was not statistically significant; however, its total indirect effect was negative (*β*=-0.45, *P* < 0.001). Among the indirect pathways, the pathway via “knowledge → vaccine confidence → vaccine hesitancy” had the strongest indirect effect (*β*=-0.27), accounting for 59.5% of the total indirect effect. The indirect effects via “knowledge → service trust → vaccine hesitancy” and “knowledge → risk perception → vaccine hesitancy” were also significant (*β*=-0.11 and − 0.07, respectively; both *P* < 0.05). The total effect of knowledge on hesitancy was negative (*β*= -0.36, *P* < 0.001) (Fig. 1).

### Physician-level recommendation practices, knowledge, and confidence

A total of 107 healthcare workers from 10 community health centers were surveyed. Recommendation enthusiasm scores (rated on a 0–4 scale, with higher scores indicating greater enthusiasm) ranged from 2.83 to 3.54 across centers, indicating a generally high level of proactive recommendation behavior (corresponding to between “often” and “always” proactively recommend). Professional knowledge scores (maximum possible score: 2) ranged from 0.43 to 1.03, suggesting an overall moderate-to-low level with notable inter-center variation (e.g., 0.43 at Tiangui Street vs. 1.03 at Qingbu). Vaccine confidence scores (rated on a 1–5 scale) ranged from 3.14 to 3.44, reflecting a moderate level between “neutral” and “somewhat agree,” with minimal between-center variation (Table 5).

**Table 5:** Summary of physician-level variables by CHC

CHC Name	Number of Physicians	Recommendation Activeness Score (Mean ± SD)	Knowledge Score(Mean ± SD)	Confidence Score(Mean ± SD)
Fenghuang	10	3.53 ± 0.23	0.80 ± 0.79	3.15 ± 0.24
Nancun	9	3.04 ± 0.74	1.00 ± 0.87	3.44 ± 0.17
Yuancun	9	3.11 ± 0.58	0.67 ± 0.87	3.17 ± 0.25
Dashi	7	2.95 ± 0.80	0.71 ± 0.49	3.14 ± 0.38
Tiangui	14	3.36 ± 0.53	0.43 ± 0.65	3.14 ± 0.31
Shiqiao	6	2.83 ± 0.28	1.00 ± 0.63	3.33 ± 0.26
Jianshebei	8	3.54 ± 0.25	0.75 ± 0.46	3.38 ± 0.23
Tangxia	11	3.33 ± 0.45	0.91 ± 0.83	3.36 ± 0.23
Qingbu	30	3.47 ± 0.32	1.03 ± 0.62	3.35 ± 0.27
Huacheng	3	3.11 ± 0.39	0.67 ± 0.58	3.33 ± 0.29

Parental-and physician‑level factors were entered into a logistic regression model with cluster‑robust standard errors. Vaccine confidence (OR: 0.21 [0.07, 0.57]) and risk perception (OR: 0.59 [0.40, 0.87]) remained protective factors against vaccine hesitancy; none of the physician‑level factors reached statistical significance. To further explore whether physician recommendation enthusiasm moderated the effect of parental confidence, we examined the interaction. In centers where physicians were less proactive in recommending the vaccine (main effect of vaccine confidence: β= -8.31), parents’ vaccination decisions were highly dependent on their own vaccine confidence. In contrast, in centers with highly proactive physician recommendations (interaction term: β = 2.10), active physician recommendations partially compensated for parents’ low confidence-suggesting that even parents with moderate confidence might choose vaccination when strongly recommended by physicians. However, this interaction effect was only marginally significant (*P* = 0.075) and should be interpreted with caution (Table 6).

**Table 6:** Multivariable logistic regression analysis of factors associated with EV-A71 vaccine hesitancy (clustered robust standard errors)

Variable	OR (95% CI)	*P* value
Confidence	0.21 (0.07, 0.57)	**0.002**
Services Trust	0.55 (0.19, 1.54)	0.252
Risk perception	0.59 (0.40, 0.87)	**0.008**
Cognition	0.77 (0.58, 1.03)	0.074
Provider recommendation activeness(Mean)	0.76 (0.45, 1.28)	0.297
Provider knowledge level(Mean)	0.60 (0.04, 8.26)	0.699
Provider confidence(Mean)	12.84 (0.60, 274.79)	0.103
Confidence × Mean provider recommendation activeness	8.16 (0.81, 82.50)	0.075

Bold values indicate statistically significant differences at *P* < 0.05

## Discussion

### Prevalence of EV-A71 vaccine hesitancy in Guangzhou

The prevalence of EV-A71 vaccine hesitancy in our sample (28.95%) falls between the 33% [11] reported in Jinan and the 50% in Chaozhou [12]. Regional income and vaccine access probably account for part of this gradient—Guangzhou’s higher disposable income [18] and more extensive CHC network may reduce financial and logistical barriers to this self‑paid vaccine, which is consistent with the observation that EV-A71 coverage is generally higher in economically developed areas [19, 20] et., a more striking comparison is with China’s National Immunization Program vaccines, where parental hesitancy is consistently below 5% [21]. That gap, even in a relatively affluent city like Guangzhou, suggests that economic factors alone cannot explain the remaining 28.95% hesitation. Instead, the perception of the vaccine’s relevance in a shifting pathogen landscape likely plays a larger role, a point we explore below.

### Sociodemographic determinants of vaccine hesitancy

Caregiver gender marked one clear divide: mothers were significantly less hesitant than fathers, a pattern also observed in a previous meta‑analysis of Chinese parents [22], and likely reflects mothers‘ greater involvement in routine childcare and health decisions. Education and occupation told a parallel story. Parents with college degrees, those working in healthcare, and those whose child had previously contracted HFMD were all less hesitant—each group having more direct or formal exposure to vaccine‑relevant information. Less‑educated and non-healthcare worker parents by contrast, are more exposed to informal narratives (media anecdotes or peer opinions) that can amplify perceived risks or downplay severity [23, 24]. Notably, household income was not significant in either bivariate or multivariable models, reinforcing that cognitive and experiential factors outweigh pure affordability in this context.

### Direct effects of confidence, trust, and risk perception

Vaccine confidence showed the strongest direct inhibitory effect on hesitancy, followed by risk perception and service trust. This hierarchy is plausible: when parents doubt vaccine safety or efficacy, they are unlikely to accept it, regardless of provider trustworthiness. This aligns with prior EV‑A71 studies that consistently identified low confidence as a key driver of hesitancy [25]. Service trust had a smaller but still significant effect. It may become decisive primarily when parents are ambivalent rather than firmly opposed, a nuance supported by Chengdu research showing that provider trust and proactive communication can facilitate EV‑A71 vaccination [26]. Risk perception, intermediate in magnitude, still independently predicted lower hesitancy. Parents who perceived HFMD as a serious threat to their own child were more willing to vaccinate [27], implying that emphasizing severe outcomes—even when EV‑A71 is no longer dominant—can still motivate protective behavior.

### Knowledge-behavior gap and mediating pathways

Only 12.7% of our respondents correctly understood that the EV-A71 vaccine remains necessary even though this serotype is no longer the dominant circulating strain. That knowledge deficit matters—but not directly. In our SEM, knowledge had no significant direct path to hesitancy; instead, its total indirect effect operated almost entirely through confidence, trust, and risk perception, with the confidence pathway accounting for 59% of that effect. Knowing the facts did not directly drive uptake; rather, facts worked by reshaping parental beliefs about vaccine safety (confidence), the credibility of providers (trust), and the personal threat of the disease (risk perception). This explains why public information campaigns alone often disappoint. They deliver knowledge, but unless that knowledge is framed to address emotional and relational concerns, it stays inert.

### Physician recommendation as a potential compensatory factor

We found a borderline interaction between physician recommendation activeness and parental vaccine confidence. The direction of the effect is suggestive: in CHCs where physicians were less proactive, parental confidence dominated the vaccination decision; where physicians were more proactive, even parents with modest confidence appeared more willing to vaccinate. This pattern is plausible—physicians’ strong endorsement may compensate for some degree of doubt, which is consistent with earlier studies on adolescent vaccines showing that enthusiastic physician recommendations facilitate uptake [14, 15]. However, the significance level is marginal, and given that we only had 10 clusters, the statistical power to detect such cross‑level moderation is modest [28]. We interpret this as hypothesis-generating, pending confirmation in a larger sample with more clusters.

### Theoretical contributions and practical implications

Our study adds to the vaccine hesitancy literature by linking an epidemiological shift (declining EV‑A71 dominance) to parental risk-benefit recalibration. It also demonstrates that the knowledge‑behavior gap is mediated by confidence more than other factor, highlighting that cognitive interventions must be paired with affective and relational strategies. Our results suggest three directions. Health communication should emphasize that EV-A71, though no longer the most common serotype, remains the most dangerous—thus correcting the “vaccine is outdated” misconception. Immunization providers should be trained not only to give facts, but to engage parents’ concerns about safety and efficacy, because that engagement builds confidence. Finally, outreach should prioritize non-healthcare worker parents, those with lower education, and those without prior HFMD experience, as these groups carry the highest hesitancy risk.

### Limitation

This study has the following limitations. First, the cross-sectional study design does not allow for causal inferences. Second, the use of convenience sampling and the inclusion of only long-term residents limit the generalizability of the findings, particularly with regard to families who have moved to the area recently. Third, instances of refusal to participate were not recorded, making it impossible to assess the response rate and nonresponse bias. Fourth, although the questionnaire was self-administered, one-on-one assistance from interviewers may have introduced social desirability bias or interviewer bias, and some variables relied on parental reports.

## Conclusions

Our findings underscore that knowledge alone does not drive EV-A71 vaccination; it works largely through confidence and trust. However, the cross-sectional design and convenience sampling limit causal inference, and the modest number of CHCs reduced power for cross-level interactions. Future studies should include more diverse regions and follow cohorts over time to capture dynamic changes in hesitancy.

## Supplementary Information

Supplementary Material 1.

Supplementary Material 2.

## Data Availability

No datasets were generated or analysed during the current study.

## Abbreviations

HFMD: Hand Foot Mouth Disease
EV-A71: Enterovirus A71
COVID-19: Coronavirus Disease 2019
KAP: Knowledge, Attitude, and Practice
CHC: Community Health Center
SEM: Structural Equation Modeling
OR: Odds Ratio
PCA: Principal Component Analysis
KMO: Kaiser-Meyer-Olkin
