# Supplementary material for: Parental EV-A71 vaccine hesitancy amid shifting HFMD pathogens: a cross-sectional study in Guangzhou
Source: BMC Public Health. 2026 Oct 19;26:3361. doi: 10.1186/s12889-026-29951-0 (PMC13756872; doi:10.1186/s12889-026-29951-0)
Supplement: Supplementary file 1 [file 12889_2026_29951_MOESM1_ESM.docx]

EV71 Vaccine (Hand, Foot and Mouth Disease Vaccine) Hesitancy Questionnaire

Dear Parent,

Greetings!

I am a staff member of the Guangzhou Center for Disease Control and Prevention, currently conducting a research project entitled *"EV71 Vaccine Hesitancy: Current Status and Influencing Factors"*. The EV71 vaccine is a new vaccine independently developed in China and was launched in 2016. It is used to prevent hand, foot and mouth disease (HFMD) caused by EV71 virus infection. The purpose of this survey is to understand public acceptance of the EV71 vaccine, to clarify concerns and doubts regarding the vaccine, and to provide evidence for improving vaccination services and communication strategies.

Your privacy is strictly protected. This questionnaire is anonymous and does not contain any information that could identify you. All completed questionnaires will be securely stored and will not be disclosed to any other individuals or institutions. Please feel free to express your honest opinions and views.

**Part 1:demographic characteristics**

Your age: _____ years

Is your child aged 5 years or under? [Single choice]

○ Yes

○ No

Your relationship to the child:[Single choice]

○ Father

○ Mother

○ Grandfather

○ Grandmother

○ Other

Your highest education level:[Single choice]

○ Middle school or below

○ High school or technical secondary school

○ Bachelor's degree or junior college

○ Master's degree or above

Average monthly household income:[Single choice]

○ < 3,000 RMB

○ 3,000–5,000 RMB

○ 5,001–10,000 RMB

○ 10,001–20,000 RMB

○ > 20,000 RMB

Your occupation:[Single choice]

○ Healthcare professional (physician, nurse, pharmacist, CDC staff, etc.)

○ Civil servant / public institution staff (non-medical)

○ Enterprise / company staff

○ Self-employed / freelancer

○ Worker / service industry staff

○ Farmer

○ Full-time homemaker

○ Retired

○ Other: _________________

Has your child ever had hand, foot and mouth disease?[Single choice]

○ Yes

○ No

Name of the community health center where you receive vaccination services: _____________

**Part 2: knowledge about HFMD and the EV-A71 vaccine**

Which of the following statements about HFMD symptoms do you think are correct? [Multiple choice]

□ Vesicles or rash appear on the hands

□ Vesicles or rash appear on the feet

□ Vesicles or ulcers appear in or around the oral mucosa

□ Vesicles appear all over the body (including the trunk and limbs)

□ Don't know

Which of the following do you think are transmission routes for HFMD?[Multiple choice]

□ Fecal–oral route (e.g., contaminated food, water, utensils)

□ Respiratory route (e.g., coughs, sneezes, droplets)

□ Close contact (e.g., contact with blister fluid or contaminated toys)

□ Blood transfusion or blood-borne transmission

□ Mosquito or insect bites

□ Don't know

Do you agree with the following statement: HFMD can be caused by multiple pathogens, including EV71.[Single choice]

○ Agree

○ Disagree

○ Don't know

Which of the following statements about severe HFMD do you think are correct?[Multiple choice]

□ Severe HFMD is mostly caused by EV71 virus

□ Severe HFMD is mostly caused by Coxsackievirus A6

□ EV71 virus infection usually causes mild symptoms and rarely leads to severe disease

□ Severe HFMD is more common in children under 5 years of age

□ Don't know

Do you agree with the following statement: severe HFMD can cause neurological symptoms.[Single choice]

○ Agree

○ Disagree

○ Don't know

Do you agree with the following statement: severe HFMD can be fatal. [Single choice]

○ Agree

○ Disagree

○ Don't know

Which virus do you know is currently the predominant cause of HFMD?[Single choice]

○ EV71 (Enterovirus 71)

○ CVA6 and CVA16 alternate as the predominant strains (Coxsackievirus A6 and A16)

○ CVA10 (Coxsackievirus A10)

○ Not sure / Don't know

Do you agree with the statement: "EV71 is no longer the predominant strain, so the EV71 vaccine is unnecessary"?[Single choice]

○ Agree

○ Disagree

○ Uncertain

Which of the following best describes your understanding of the protective scope of the EV71 vaccine?[Single choice]

○ It can prevent all types of HFMD

○ It only prevents HFMD caused by the EV71 virus

○ It has no preventive effect against any type of HFMD

○ I am not sure what types it can prevent

Do you agree with the following statement: the EV71 vaccine should be given as early as possible once the child reaches the eligible age.[Single choice]

○ Agree

○ Disagree

○ Don't know

Are you aware that the EV71 vaccine provides nearly 100% protection against severe HFMD?[Single choice]

○ Yes

○ No

**Part 3: attitudes**

In your opinion, how high is the risk of hand, foot and mouth disease infection among children aged 5 years and under in Guangzhou? [Single choice]

○ Very high

○ Relatively high

○ Moderate

○ Relatively low

○ Very low

Are you worried that your child might develop severe complications (e.g., encephalitis, pulmonary edema, myocarditis) from HFMD infection?[Single choice]

○ Very worried

○ Somewhat worried

○ Moderate

○ Not very worried

○ Not worried at all

Are you concerned about potential adverse reactions to the EV71 vaccine?[Single choice]

○ Very worried

○ Somewhat worried

○ Moderately worried

○ Not very worried

○ Not worried at all

You believe the EV71 vaccine is safe.[Single choice]

○ Strongly agree

○ Agree

○ Neutral

○ Disagree

○ Strongly disagree

You believe the EV71 vaccine can prevent HFMD. [Single choice]

○ Strongly agree

○ Agree

○ Neutral

○ Disagree

○ Strongly disagree

You believe the EV71 vaccine can reduce severe cases and deaths from HFMD. [Single choice]

○ Strongly agree

○ Agree

○ Neutral

○ Disagree

○ Strongly disagree

If your child receives the EV71 vaccine, they can be protected against severe HFMD, but they may still contract mild HFMD caused by other viruses. Can you accept this? [Single choice]

○ Strongly accept

○ Accept

○ Neutral

○ Not accept

○ Strongly not accept

Do you trust the vaccine-related information provided by vaccinators, experts, and other professionals? [Single choice]

○ Strongly trust

○ Somewhat trust

○ Moderate

○ Somewhat distrust

○ Strongly distrust

Do you trust the vaccinators? [Single choice]

○ Strongly trust

○ Somewhat trust

○ Moderate

○ Somewhat distrust

○ Strongly distrust

Do you agree with the statement: "Vaccinators recommend vaccines primarily for the child's health, rather than to meet targets or generate profit"? [Single choice]

○ Strongly agree

○ Agree

○ Neutral

○ Disagree

○ Strongly disagree

Are you willing to have your child vaccinated against EV-A71?[Single choice]

○ Yes (already vaccinated or planning to vaccinate)

○ Uncertain

○ No, I refuse vaccination

End of Questionnaire

Thank you for your participation!
