# Supplementary material for: Parental EV-A71 vaccine hesitancy amid shifting HFMD pathogens: a cross-sectional study in Guangzhou
Source: BMC Public Health. 2026 Oct 19;26:3361. doi: 10.1186/s12889-026-29951-0 (PMC13756872; doi:10.1186/s12889-026-29951-0)
Supplement: Supplementary file 2 [file 12889_2026_29951_MOESM2_ESM.docx]

Questionnaire on EV71 Vaccine Recommendation Behaviors among Healthcare Workers at Community Health Centers

Dear Doctor,

Greetings!

I am a staff member of the Guangzhou Center for Disease Control and Prevention, currently conducting a research project entitled *"EV71 Vaccine Hesitancy: Current Status and Influencing Factors"*. The EV71 vaccine is a new vaccine independently developed in China and was launched in 2016. It is used to prevent severe hand, foot and mouth disease (HFMD) caused by EV71 virus infection. The purpose of this survey is to understand vaccinators' attitudes toward recommending the EV71 vaccine and the influencing factors, in order to optimize vaccination services. This questionnaire is anonymous and the data collected will be used solely for statistical analysis. Thank you for your cooperation!

1. Your workplace (institution):_____________

2. Your contact information: _____________

3. Do you directly participate in vaccine recommendation or vaccination services at the immunization clinic?

① Yes

② No *(End of survey)

4. Gender:

① Male

② Female

5. Age:

① ≤30 years

② 31–40 years

③ 41–50 years

④ >50 years

6. Years of experience in vaccination services:

① <5 years

② 5–10 years

③ 11–20 years

④ >20 years

7. Professional title:

① Junior

② Intermediate

③ Senior or above

8. How effective do you consider the EV71 vaccine in preventing severe HFMD?

① Very effective (>90%)

② Moderately effective (70%–90%)

③ Fairly effective (50%–70%)

④ Poorly effective (<50%)

⑤ Not sure

9. Overall, how do you evaluate the safety of the EV71 vaccine?

① Very safe

② Relatively safe

③ Moderate

④ Somewhat unsafe

⑤ Very unsafe

10. Overall, do you agree that "age-eligible children should be routinely vaccinated with the EV71 vaccine"?

① Yes

② No

11. Do you know which virus strain is currently the most prevalent cause of HFMD?(Single choice)

① EV71 (Enterovirus 71)

② CVA6 (Coxsackievirus A6)

③ CVA16 (Coxsackievirus A16)

④ Not sure / Don't know

12. Based on the current pathogen spectrum shift, how do you think the necessity of recommending the EV71 vaccine compares with that of five years ago?

① Much higher than five years ago

② About the same as five years ago

③ Somewhat lower than five years ago

④ Much lower than five years ago

13. In your daily work, how often do you proactively recommend the EV71 vaccine to parents of age-eligible children?

① Always (>90%)

② Often (50%–90%)

③ Sometimes (10%–50%)

④ Rarely or never (<10%)

14. What is your most common approach to recommending the EV71 vaccine? (Single choice, select the primary method)

① Proactively inform parents "recommended" and explain the value of preventing severe disease

② Casually ask "Do you want it?"

③ Only respond when parents ask proactively

④ Distribute brochures for parents to read themselves

⑤ Do not bring it up

15. What is the main barrier that prevents you from recommending the EV71 vaccine more actively? (Single choice)

① EV71 is no longer the predominant strain, so I lack confidence in my recommendation

② The vaccine is expensive, and I am concerned about the financial burden on parents

③ Frequent refusal by parents affects my motivation

④ Some parents have reported that "the child still got the disease after vaccination," which has reduced my confidence in the vaccine

⑤ Concern about adverse reactions leading to disputes

⑥ Too busy in the clinic, no time to provide detailed explanations

⑦ Insufficient understanding of the latest pathogen dynamics, afraid of making mistakes

⑧ No performance appraisal requirement for recommendation, so it does not matter if I do not recommend

⑨ Other: _________________

16. Which of the following would most effectively promote your active recommendation?(Single choice)

① Provision of standard communication scripts on "how to discuss the pathogen spectrum shift in the context of vaccine recommendation"

② Provision of local data on the effectiveness of EV71 vaccine in preventing severe disease

③ Lower vaccine prices or inclusion in the free vaccination program

④ Inclusion of recommendation in performance appraisal

⑤ Reducing clinic workload to ensure adequate communication time

⑥ Provision of more health education materials for parents

17. When communicating with parents, do you actively emphasize the value of the EV71 vaccine in preventing severe disease?

① Always / Often

② Sometimes

③ Rarely / Never

End of Questionnaire

Thank you for your support!
